# Plasmablastic lymphoma masked by hidradenitis suppurativa

**DOI:** 10.1016/j.jdcr.2022.06.016

**Published:** 2022-07-03

**Authors:** Sarah Preis, Alphina Kain, Tilo Biedermann, Thomas Volz

**Affiliations:** Department of Dermatology and Allergy, Technical University of Munich, School of Medicine, Munich, Germany

**Keywords:** chronic inflammation, hematologic malignancyz, hidradenitis suppurativa, lymphoma, HS, hidradenitis suppurativa, IL, interleukin, PBL, plasmablastic lymphoma

## Introduction

Hidradenitis suppurativa (HS) is a chronic inflammatory skin disease associated with an elevated overall and specific risk of cancer. In addition to a risk for developing solid malignancies like squamous cell carcinoma, patients with HS bear an increased risk for the occurrence of hematological diseases like non-Hodgkin lymphoma, Hodgkin lymphoma, and cutaneous T-cell lymphoma.^1^^,^^2^ Patients with HS develop malignant lymphomas more frequently than members of the general population, although the specific mechanisms between chronic inflammatory conditions and neoplastic transformation remain mostly unknown.^2^

## Case report

**Fig 1:**
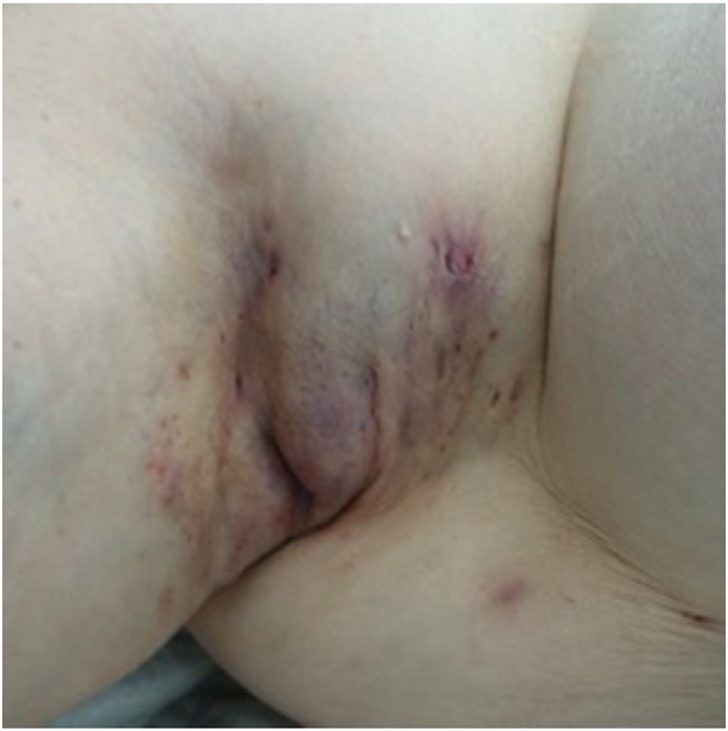
Right axilla. A firm conglomerate of erythematous, pressure-indolent nodules and fistulas was found in the right axilla.

A 56-year-old female patient with a 20-year history of Hurley stage III HS presented with worsening of her right axillary skin lesions over the last 2 months (Fig 1). The patient's past medical history also included a cholecystectomy, hysterectomy, and drug-controlled arterial hypertension. On physical examination, she had numerous inflammatory nodules, fistulas, and bridle scars in the bilateral axillae, submammary folds, and gluteal region.

**Fig 2:**
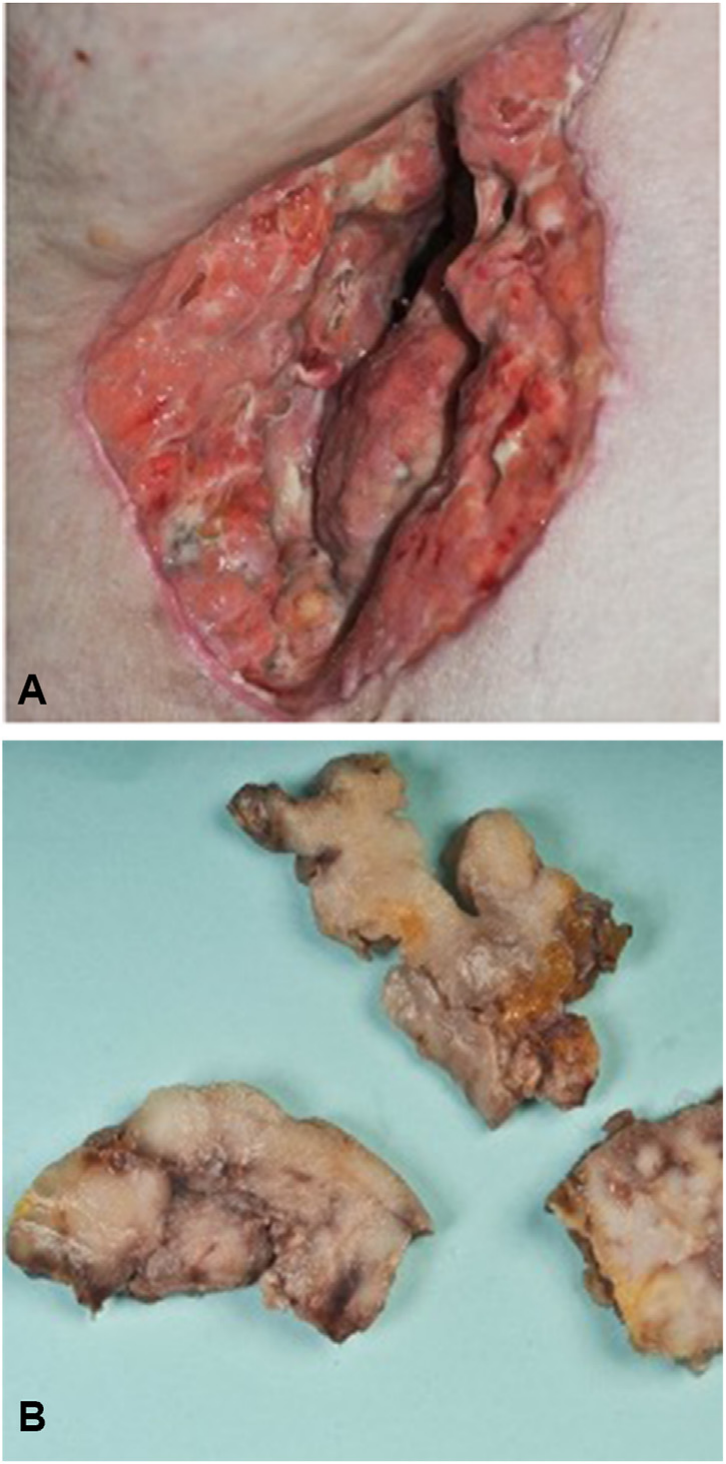
**A**, Right axilla. Operation field after removal of a large *brownish* tumor mass. **B**, Macroscopically, the resection showed *brownish-yellow* nodular tissue.

**Fig 3:**
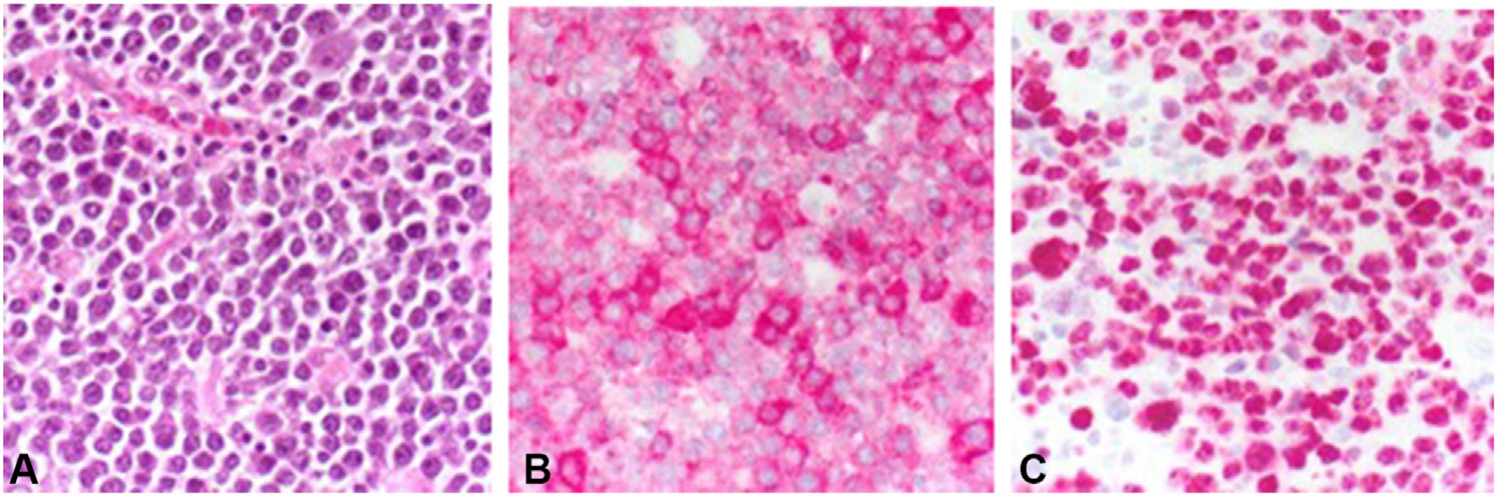
The histology showed fibrotic inflammation and a tumorous infiltrate with moderately pleomorphic tumor cells, which strongly expressed EMA and were negative for PanCK, ChromgrA, CEA, CK20, MelanA, S100, and TTF-1 (**A**: HE, **B**: EMA, **C**: KI-67).

Over the last 2 months, the patient reported a significant worsening of the right axillary skin lesions (Fig 1). Previously the patient was treated systemically with high-dose vitamin A, but due to elevations in liver values, this therapy was discontinued. The patient was started on antibiotic therapy with clindamycin and rifampicin; however, this regimen failed to alleviate her symptoms. Due to the axillary pain, the restriction of movement from existing scars, and the lack of response from the antibiotic therapy, a decision was made to proceed with a wide local excision of the affected area. During surgery, a 10 × 15-cm brownish tumor mass nearly filling the entire right axilla was found in the subcutaneous tissue alongside substantially fibrotic tissue interspersed with fistula tracts (Fig 2, *A* and *B*). Histology showed moderately pleomorphic, plasmacytic cells with small- to medium-sized nuclei that were intermixed with multinucleated cells. The cells strongly expressed EMA, MUM1, and CD138(Fig 3, *A*–*C*). The proliferation index Ki-67 was nearly 90%. Stains for pancytokeratin, chromogranin, CEA, CK20, CD3, MelanA, S100, and TTF-1 were negative. Upon further inquiry, the patient also reported having pronounced B-symptoms, including increased night sweats, a weight loss of 7 kg in 6 weeks, and general weakness. Repeat physical examination revealed an enlarged submandibular lymph node, and further lab testing revealed a leukocytosis with an accompanying left shift. A computed tomography scan showed lymphadenopathy in the right submandibular region, bilateral axillae, and the bilateral subpectoral region. A bone marrow biopsy of the iliac crest was performed, and cytology showed large cells with partially amorphous, medium-sized nuclei, and nucleoid-like inclusions. Lesional cells were strongly positive for CD138. A kappa light chain restriction was present, and EBER-ISH was positive. Stains for CD20, PAX5, CD5, TdT, and CD34 were negative. Considering the findings, the diagnosis of a plasmablastic lymphoma (PBL), Ann-Arbor stage IVB, was made. The patient was started on chemotherapy according to the cyclophosphamide, doxorubicin, etoposide, vincristine and prednisone protocol with additional administration of bortezomib. To date, 2 years later, the patient is in full remission.

## Discussion

There is increasing evidence that HS patients are prone to developing hematological malignancies, with HS being associated with a 2- to 4-fold increased lifetime risk of lymphoma development.^2^ Although a pathophysiological link has not yet been identified, it is assumed that the aberrant immune response present in HS may be the driving force for lymphoma development. In HS, the immune defense does not suffice in containing the inflammation but rather leads to a systemic inflammatory response.^3^ Thus, increased concentrations of tumor necrosis factor-alpha, interleukin (IL)-1, IL-17, and IL-23 are detectable in the serum of HS patients.^4^ Elevated levels of proinflammatory substances have a decisive influence on various pathophysiological developments. For instance, higher levels of IL-17 and decreased levels of anti-inflammatory IL-10 are associated with reduced expression of antiapoptotic and proliferative genes, which may promote tumor development.^5^ The systemic inflammation in HS can lead to inflammation triggered T-cell blast proliferations, as described in a 35-year-old patient with a long history of HS, who showed a benign intralymphatic proliferation of T-cell lymphoid blasts in the affected area of the groin.^6^ Moreover, a 27-year-old man, suffering from a high inflammatory load with comorbidities of vitiligo, psoriasis, and HS, developed a T-cell/histiocyte-rich large B-cell lymphoma in his retroperitoneal and inguinal lymph nodes.^7^

PBL is a rare, very aggressive non-Hodgkin lymphoma and is a subtype of diffuse large B-cell lymphoma.^6^ PBL most commonly occurs in the oral cavity, but it may also arise in the lymph nodes, soft tissue, stomach, and lungs.^8^ It is commonly associated with HIV infection.^9^ In the minority of HIV-negative patients with PBL, associations with chronic inflammatory (Crohn’s disease), immunosuppression (post-transplantation), or autoimmune diseases are described.^8^^,^^10^^,^^11^ After diagnosis of PBL, our patient underwent extensive testing to screen for underlying diseases. HIV testing was negative. Colonoscopy and gastroscopy, a gynecological screening, and extensive blood testing were performed, whose results were not indicative of an underlying disease. As the prognosis is dismal (median overall survival of 9 m, 2-year-overall survival rate of 10%), there is no standard care for patients with PBL. Cyclophosphamide, doxorubicin hydrochloride (hydroxydaunorubicin), vincristine sulfate (Oncovin), and prednisone as well as other forms of chemotherapy have been reported.^8^ Recent studies with the proteasome inhibitor bortezomib showed promising results for plasmablastic lymphomas, which is why bortezomib was administered for our patient.^12^

With chronic inflammatory skin diseases being increasingly recognized as inflammatory systemic diseases, attention should be given to the associated development of hematologic neoplasms. Especially in the groin and axilla, the typical predilections sites of HS, clinical signs of hematological malignancies such as lymph node swelling may be easily overlooked. As severe HS leads to substantial morphological alterations of the skin and subcutaneous tissue, lymphoma can present as an HS “look-alike,” morphologically mimicking deep-seated HS lesions as demonstrated by our patient.

## Conflict of interest

Tilo Biedermann gave advice to or received an honorarium for talks or research grants from the following companies: Alk-Abelló, Celgene-BMS, Galderma, GlaxoSmithKline, Leo Pharma, Lilly Deutschland GmbH, Mylan, Novartis, Phadia-Thermo Fisher, Sanofi-Genzyme, Regeneron, and Viatris. T. Volz gave advice to or received an honorarium for talks or research grants from Abbvie, Allmiral, La Roche Posay, Hipp, and Novartis. S. Preis received an honorarium for talks from Janssen.
